# Continuous monitoring of relative blood volume allows real-time assessment of intradialytic hypotension risk

**DOI:** 10.1093/ckj/sfag052

**Published:** 2026-02-19

**Authors:** Julien Aniort, Thomas Bachelet, Pascal Seris, Thibault Dolley-Hitze, Marc Bouiller, Camilia Beji, Valerie Batel, Bruno Pereira, David Attaf, Pascal Kopperschmidt, Anne-Elisabeth Heng, Bernard Canaud

**Affiliations:** Department of Nephrology, Dialysis and Transplantation, Gabriel Montpied Hospital, CHU Clermont-Ferrand, Clermont-Ferrand, France; Human Nutrition Unit, INRAE UMR 1019, Clermont Auvergne University, Clermont-Ferrand, France; Clinique Saint-Augustin (CTMR), ELSAN, Bordeaux, France; Service de Néphrologie-Dialyse, AURA Paris Plaisance, Paris, France; Fondation AUB Santé, Unité de dialyse, Saint-Malo, France; Centre Hospitalier Emile Roux, Le Puy-en-Velay, France; Department of Nephrology, Dialysis and Transplantation, Gabriel Montpied Hospital, CHU Clermont-Ferrand, Clermont-Ferrand, France; Department of Nephrology, Dialysis and Transplantation, Gabriel Montpied Hospital, CHU Clermont-Ferrand, Clermont-Ferrand, France; Biostatistics Unit, CHU Clermont-Ferrand, Clermont-Ferrand, France; Fresenius Medical Care, Fresnes, France; Fresenius Medical Care, Global Research & Development, Bad Homburg, Germany; Department of Nephrology, Dialysis and Transplantation, Gabriel Montpied Hospital, CHU Clermont-Ferrand, Clermont-Ferrand, France; Human Nutrition Unit, INRAE UMR 1019, Clermont Auvergne University, Clermont-Ferrand, France; Montpellier University, UFR Medicine, Montpellier, France

**Keywords:** blood pressure, hemodialysis, intradialytic hypotension, machine learning, relative blood volume

## Abstract

**Background:**

Intradialytic hypotension (IDH) is a frequent complication of hemodialysis, associated with patient discomfort, end-organ injury, and higher mortality. Prediction remains challenging. The value of relative blood volume (RBV) monitoring is debated. We investigated whether RBV indicates IDH risk and enables real-time prediction.

**Methods:**

In this prospective, multicenter study, 56 patients across 459 hemodialysis sessions were monitored with RBV sensors. IDH was defined as a systolic blood pressure <90 mmHg with a ≥20 mmHg decrease from baseline. RBV trajectories were analyzed using unsupervised clustering to identify risk patterns, and a time-varying RBV threshold was derived. Associations with IDH were tested using generalized linear mixed models (GLMMs) with patient as random effects. Predictive performance was evaluated using GLMMs and benchmarked against XGBoost, with validation performed session wise and patient wise.

**Results:**

IDH occurred in 29.7% of sessions and was associated with RBV decline. At the time of measurement, each 1% decrease in RBV increased IDH risk (OR = 1.05; *P* < .001). Falling below the dynamic RBV threshold doubled the odds of IDH (OR = 2.37; *P* < .001). In predictive analysis assessing IDH occurrence within the subsequent 10–60 minutes, GLMMs showed good discrimination in session-wise validation (AUC = 0.77) but were less accurate in patient-wise validation (AUC = 0.62). Incorporating patient-specific features improved patient-wise performance (AUC = 0.85 for GLMM; 0.86 for XGBoost).

**Conclusions:**

RBV decline is a robust predictor of IDH. Including RBV in real-time risk models may help personalize ultrafiltration strategies and improve dialysis safety.

## Graphical Abstract

**Graphical Abstract:**
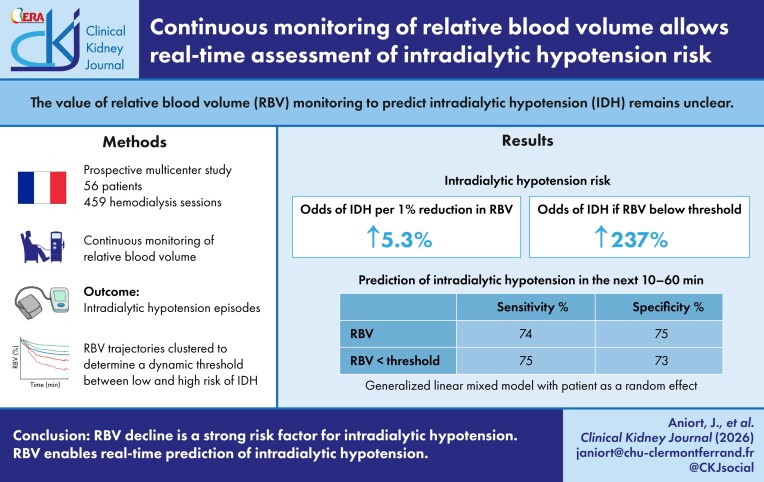

KEY LEARNING POINTS **What was known:**

- Intradialytic hypotension (IDH) arises from hypovolemia, but hemodynamic responses are highly variable between patients.
- The relationship between relative blood volume (RBV) changes and IDH has been studied, but results have been inconsistent and sometimes contradictory.
- No study has evaluated the utility of continuous RBV monitoring to predict IDH.

**This study adds:**

- RBV decline is strongly associated with the occurrence of IDH.
- Crossing below a time-dependent RBV threshold significantly increased the risk of IDH.
- Incorporating patient effect improved the performance of RBV-based prediction models.

**Potential impact:**

- Integrating RBV monitoring data could enable earlier identification of patients at imminent risk of IDH.
- Such models could support personalized fluid removal strategies during dialysis sessions.
- This approach may reduce IDH-related complications and improve patient outcomes in routine dialysis practice.

## INTRODUCTION

Intradialytic hypotension (IDH) remains a major concern in the management of patients undergoing hemodialysis (HD). It is among the most frequent complications during dialysis, occurring in ∼5%–30% of sessions [1].

Beyond the immediate symptoms it causes—such as fatigue, dizziness, or malaise—IDH contributes to repeated episodes of tissue hypoperfusion, which may progressively impair organ function. It has been associated with myocardial stunning and impaired systolic performance [2], accelerated cognitive decline [3], increased risk of mesenteric ischemia bacterial translocation [4], loss of residual kidney function [5], and higher incidence of vascular access thrombosis [6], ultimately contributing to increased mortality in this population [7, 8].

Historically, IDH has been defined as a drop in systolic blood pressure (SBP) >20 mmHg or MAP >10 mmHg, particularly when accompanied by symptoms or requiring clinical intervention. A *post hoc* analysis of the Hemodialysis (HEMO) Study, along with real-world data from large cohorts analyses [8], has challenged this conventional criteria by demonstrating that only an absolute nadir SBP <90 mmHg was independently associated with increased mortality, regardless of the presence of symptoms [7]. These findings contributed to a shift toward more objective, outcome-oriented definitions of IDH. This definition of IDH has been validated by the recent KDIGO controversies conference [9].

Ultrafiltration is the primary physiological driver of IDH [10]. Under normal conditions, this volume loss is compensated by vascular refilling from the interstitial space [11]. When ultrafiltration exceeds the body’s refilling capacity, blood volume and cardiac output decrease [12]. This reduction triggers a compensatory sympathetic response aimed at maintaining blood pressure. When impaired, IDH [13] can occur because of decrease of cardiac output and/or vascular resistance [14]. Several risk factors for IDH have been reported some linked to patient profile such as cardiac dysfunction [15], autonomic neuropathy [16], hypoalbuminemia [17], while others are related to practices, the use of antihypertensive medications [18], dialysate sodium [19], and temperature [20].

Relative blood volume (RBV)—defined as the percentage change of blood volume at a given time during the session to its baseline value—is commonly estimated through continuous hematocrit monitoring. This provides a surrogate for real-time changes in intravascular volume. Several observational studies have investigated the association between RBV trends and IDH risk [12, 21–24]. However, most studies have failed to establish a clear relationship between RBV decline and the risk of IDH, and to identify a reproducible RBV threshold predictive of IDH. Moreover, they mostly relied on single timepoint RBV measurements, and none accounted for continuous RBV monitoring throughout the hemodialysis session.

In an effort to prevent IDH, a protocolized manual reduction of ultrafiltration rates based on RBV values was associated with unexpected increased hospitalization and mortality rates [25]. Various automatic ultrafiltration biofeedback systems, which continuously adjust UF rates in real time based on RBV monitoring, have been developed. These systems operate based on empirically derived RBV trajectories or threshold values. While initial studies in small cohorts suggested potential benefits [26], subsequent randomized controlled trials failed to demonstrate consistent improvements in clinical outcomes, including morbidity and survival [27].

The objective of this study was to investigate the association between RBV and IDH and to assess the potential value of RBV monitoring for real-time prediction of hypotensive events during dialysis.

## MATERIALS AND METHODS

### Study design and population

A prospective, observational, multicenter study was conducted across five French hemodialysis centers between January 2016 and December 2023. Eligible participants were adults aged ≥18 years with end-stage renal disease undergoing maintenance HD or online hemodiafiltration (HDF) for at least 3 months, receiving treatment three times per week with session durations of 3–5 hours. Inclusion also required a history of at least two episodes of IDH in the preceding month. Exclusion criteria were: hemoglobin levels <7 or >15 g/dl, ongoing acute illness, or a life expectancy of <6 months. The study was approved by the French Research Ethics Committee Sud-Est VI (AU-1158) and registered on ClinicalTrials.gov (NCT03350308). Written informed consent was obtained from all participants.

### Dialysis protocol

Dialysis sessions were performed using Fresenius Medical Care 5008 or 6008 machines. For patients treated with online HDF, substitution fluid was administered in post-dilution mode, regulated by the Autosub + control function. Conductivity profiles were not used, and sessions were conducted under isothermic dialysis conditions [28]. Dialysate composition, dialysis membrane, blood and dialysate flow rates, anticoagulation protocols, and prescribed dry weight were left to the discretion of the attending nephrologist. No specific protocol-driven adjustments were imposed. Notably, the prescribed dry weight did not necessarily match the dry weight estimated by bioimpedance spectroscopy performed as part of this study (see next). Dry weight could be adjusted during the observation period. Blood pressure and heart rate were measured every 30 minutes via oscillometric methods and when indicated by the care team. IDH episodes were managed according to standard clinical practice, including Trendelenburg positioning, immediate cessation of ultrafiltration, and saline infusion when required. No vasopressors or preventive measures were used.

### Data collection

Baseline patient data included age, sex, weight, height, dialysis vintage, comorbidities (including heart failure, ischemic heart disease, valvular disease, atrial fibrillation, and peripheral artery disease), medication use, serum albumin, and hemoglobin levels (measured within one month prior to inclusion). Body composition was assessed using multifrequency bioimpedance spectroscopy at the first dialysis session (Body Composition Monitor, Fresenius Medical Care), providing measurements of total body water as well as intracellular and extracellular water volume, fluid volume overload, normovolemic status, lean tissue and fat tissue index. Blood pressure and heart rate were measured every 30 minutes via oscillometric methods and when clinically indicated by the care team.

During nine consecutive hemodialysis sessions per patient, prescribed dry weight and actual pre- and post-dialysis body weight were recorded. Dialysis machine data were extracted at 1-minute intervals and included dialysate sodium and bicarbonate concentrations, blood and dialysate flow rates, dialysate temperature, systolic and diastolic blood pressure, heart rate, K*t*/*V* and serum sodium measured by ionic dialysance and hematocrit measured by the blood volume monitor. RBV was calculated as: Ht(0)/H(*t*) where Ht(0) is the hematocrit at baseline and H(*t*) is the hematocrit at time *t*.

### Definition of intradialytic hypotension

IDH was defined as a SBP <90 mmHg with a decrease in SBP of <20 mmHg from the first recorded measurement of the session. This definition was chosen because a nadir SBP <90 mmHg has been validated by KDIGO [9], and the initial pre-dialysis blood pressure value is not influenced by dialysis-related interventions.

### Statistical analysis

Statistical analyses were performed using R v.4.5. Continuous variables were summarized as mean ± SD or median (IQR), based on Shapiro–Wilk normality test results, and categorical variables as counts and percentages. IDH frequency across RBV categories was compared using Fisher’s exact test. RBV values between hypotensive and non-hypotensive periods were compared using the Wilcoxon rank-sum test. Associations between RBV and IDH were assessed using generalized linear mixed models (GLMMs) with patient as a random effect.

RBV trajectories were clustered and a time-dependent RBV threshold was determined by identifying, at each timepoint, the value that maximized the Youden index for distinguishing high- versus low-risk clusters. The difference between post-dialysis weight minus BCM-derived dry weight between high and low-risk cluster, were compared using the Wilcoxon rank-sum test. The timing of hypotensive events between clusters was evaluated by comparing event frequencies at each 30-minute interval using Fisher’s exact test. The association between cluster or crossing below the dynamic threshold and IDH risk was assessed using GLMMs

Predictive models for IDH within 10–60 minutes were developed using GLMMs and a regularized gradient boosting algorithm (XGBoost), with patient-level and session-level validation. For patient-level validation, GLMM predictions on the test set were obtained using only the fixed effects, as patient-specific random effects cannot be estimated for new, unseen patients. Model discrimination was evaluated by the area under the ROC curve (AUC), sensitivity, and specificity. Detailed analytical procedures, preprocessing steps, and model specifications are provided in the Supplementary Data.

## RESULTS

### Population and baseline characteristics

Among the 60 patients initially enrolled, one died before the study began and another withdrew consent. Hemodialysis machine data were unavailable for 40 sessions, including two patients with no usable sessions. The final analysis included 459 hemodialysis sessions from 56 patients. Baseline characteristics are summarized in Table 1. The mean age was 72 years, with an equal distribution of male and female participants. Cardiovascular comorbidities were common. Most patients were prescribed antihypertensive treatment. Characteristics of the dialysis sessions are provided in Table 2. IDH occurred in 29.7% of sessions.

**Table 1:** Baseline characteristics of the study population.

Characteristics	*n* = 56
Sex (female), *n* (%)	28 (50.0)
Age (years)	72.2 ± 11.5
Hypertension, *n* (%)	50 (89.3)
Diabetes, *n* (%)	30 (53.6)
Heart failure, *n* (%)	8 (14.3)
Ischemic heart disease, *n* (%)	18 (32.1)
Valvular disease, *n* (%)	8 (14.3)
Atrial fibrillation, *n* (%)	14 (25.0)
Peripheral artery disease, *n* (%)	11 (19.6)
LVEF (%)	59.6 ± 11.3
Dialysis vintage (months)	113.9 ± 461.3
Height (m)	1.6 ± 0.1
Dry weight (kg)	76.7 ± 19.5
On antihypertensive treatment, *n* (%)	33 (58.9)
Number of antihypertensives treatment, *n*	0.8 ± 0.9
Serum sodium (mmol/l)	136.8 ± 3.4
Albumin (g/l)	37 ± 5
Hemoglobin (g/dl)	11.5 ± 1.3
FTI (kg/m)	16.1 ± 7.3
LTI (kg/m)	12.3 ± 4.7

FTI, fat tissue index; LVEF, left ventricular ejection fraction; LTI, lean tissue index.

**Table 2:** Dialysis parameters of the study sessions.

Dialysis parameters	(*n* = 459)
Blood flow rate (ml/min)	355.1 ± 50.9
Dialysate flow rate (ml/min)	511.1 ± 101.4
Dialysate temperature (°C)	36.4 ± 0.2
Dialysate sodium (mmol/l)	139.4 ± 1.7
Dialysate bicarbonate (mmol/l)	33.3 ± 1.4
Pre-dialysis SBP (mmHg)	128.8 ± 24.4
Post-dialysis SBP (mmHg)	114.0 ± 24.1
Session duration (min)	243.2 ± 24.7
K*t*/*V*	1.44 ± 0.45
Ultrafiltration (l)	2.3 ± 1.1
Ultrafiltration (% body weight)	3.1 ± 1.3
Ultrafiltration rate (ml/h/kg)	7.6 ± 3.1

### A decrease in relative blood volume is a risk factor for intradialytic hypotension

**Figure 1:**
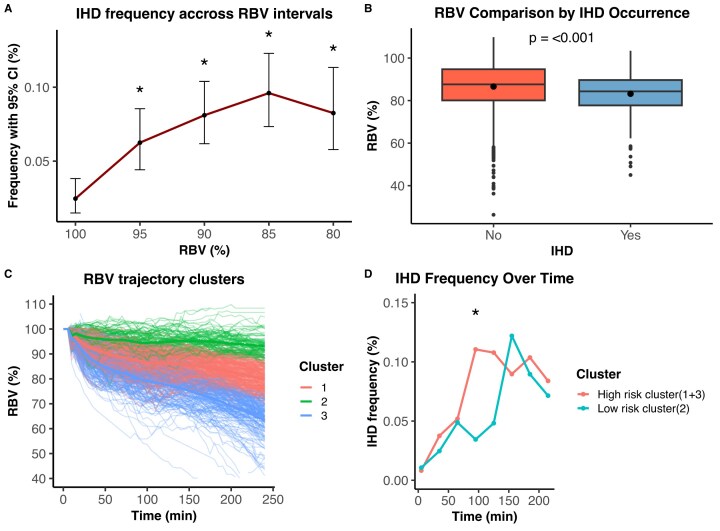
Association between RBV and intradialytic hypotension (IDH). (a) Incidence of IDH stratified by RBV categories. Lower RBV values were associated with a higher frequency of hypotensive events. Asterisks indicate statistically significant differences compared with the reference category (RBV ≥95%) (*P* < .05, Wilcoxon test). (b) Distribution of RBV values during hypotensive and non-hypotensive periods, with lower RBV observed during hypotensive episodes. (c) Classification of hemodialysis sessions into three clusters based on RBV trajectories using dynamic time warping. Clustering was performed at the session level and each curve represents one hemodialysis session. (d) Cumulative incidence of IDH during the session according to RBV cluster groups. A significant difference between high-risk and low-risk clusters was observed at 90 minutes (*P* < .05, Fisher’s exact test).

The incidence of IDH increased as RBV decreased. Compared with the reference interval (RBV ≥95%), the frequency of hypotensive events was significantly higher in lower RBV categories, peaking in the 85–90% range (8.1%) and remaining elevated below 85%. This trend was statistically significant (*P* < .05 for each lower category) (Fig. 1a). Furthermore, RBV values were also significantly lower during hypotensive episodes compared to non-hypotensive periods [median (IQR): 84.3 (78.0–90.3) vs. 87.6 (81.4–93.7), *P* < .001] (Fig. 1b). In a GLMM, RBV was then analyzed as a time-varying predictor. Each 1% decrease in RBV (i.e. a 1-point increase in ΔRBV = 100–RBV) was associated with a 5.3% increase in the odds of IDH at the same time (OR = 1.053; 95% CI: 1.037–1.069; *P* < .001).

### Relative blood volume trajectories identify sessions at higher risk of hypotension

Sessions were grouped into three clusters based on their dynamic RBV trajectories (Fig. 1c). The incidence of IDH varied significantly by cluster: 33.2% in cluster 1, 30.9% in cluster 3 and 18.8% in cluster 2. Clusters 1 and 3 were thus combined into a “high-risk” group, while cluster 2 served as the “low-risk” reference. The corresponding final RBV, ultrafiltration volume (arrival weight minus post-dialysis weight), and dry-weight estimation discrepancy (prescribed dry weight minus BCM-derived dry weight) are shown in Table 3. In a GLMM, sessions in the high-risk cluster were associated with significantly higher odds of IDH compared to low-risk sessions (OR = 2.58; 95% CI: 1.19–5.58; *P* = .017). Temporal analysis showed that high-risk sessions not only exhibited a greater overall frequency of hypotension, but also developed these events earlier during dialysis session. A divergence in event frequency between groups was already evident before the 90-minute mark, with a significant difference observed at 90 minutes (*P* < .05, Fisher’s exact test) (Fig. 1d).

**Table 3:** Final relative blood volume (RBV), ultrafiltration volume (arrival weight minus post-dialysis weight), and dry-weight estimation discrepancy.

	Low-risk cluster sessions*n* = 85	High-risk cluster sessions*n* = 374	*P*
Final RBV (%)	92.1 [86.4 to 97.8]	71.5 [56.5 to 86.5]	<.001
Ultrafiltration (l)	1.5 [0.2 to 2.8]	2.4 [1.0 to 3.7]	<.001
Prescribed dry weight, BCM-derived dry weight (kg)	0.0 [−2.4 to 2.4]	−0.9 [2.6 to 1.7]	.003

### Crossing below the time-dependent relative blood volume threshold is a significant risk factor for intradialytic hypotension

**Figure 2:**
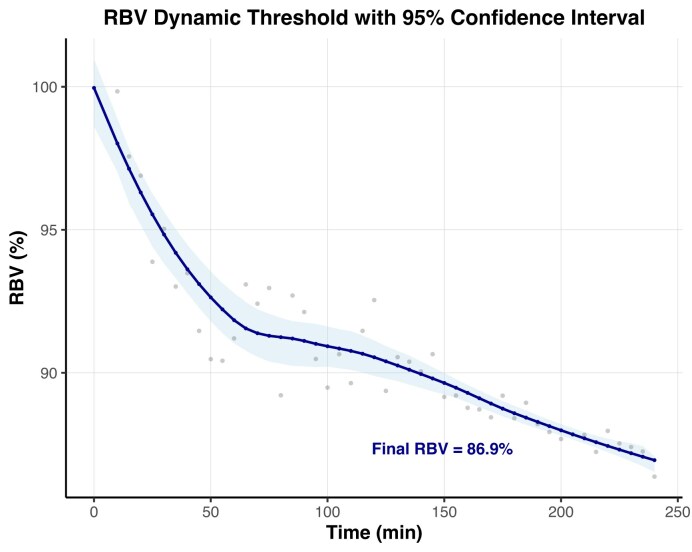
Dynamic RBV threshold. A time-dependent RBV threshold was derived by identifying, at each timepoint, the value that maximized the Youden index for distinguishing low and high-risk cluster for IHD. The resulting threshold curve was smoothed using locally estimated scatterplot smoothing (LOESS). The LOESS-smoothed curve is displayed with 95% bootstrap confidence intervals, and the raw (non-smoothed) threshold values are plotted as gray points.

To better reflect time-varying risk, a time-dependent threshold of RBV was computed for distinguishing low and high-risk cluster (Fig. 2 ). Crossing below this dynamic RBV threshold was strongly associated with increased hypotension risk at the same time. A GLMM found that being below the threshold was associated with a >2-fold increase in the odds of IDH (OR = 2.37; 95% CI: 1.65–3.38; *P* < .001).

**Figure 3:**
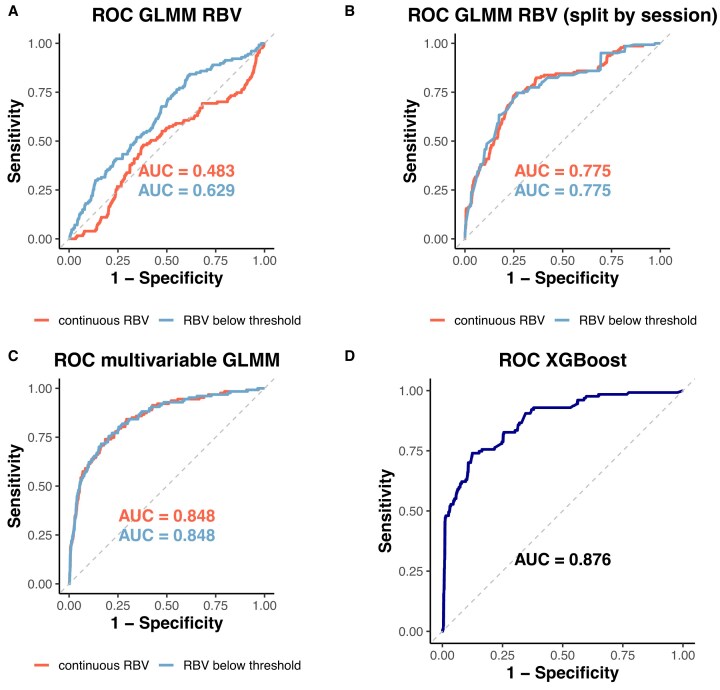
Predictive performance of models for future intradialytic hypotension (IDH). (a) Receiver operating characteristic (ROC) curves of a GLMM trained using RBV to predict hypotension in the subsequent 10–60 minutes. Model performance is shown for training and test sets using a patient-based data split. (b) ROC curves of the same GLMM evaluated using a session-based data split. (c) ROC curves comparing the multivariable GLMM (including RBV, its temporal derivative, SBP, ΔSBP, and HR) with a simplified binary model using only the ‘RBV below dynamic threshold’ indicator. Both models were evaluated on the test set. (d) Performance of a regularized XGBoost classifier, trained using cross-validation stratified by patient. ROC curve shown on the independent test set.

### RBV-based model can predict imminent intradialytic hypotension

To predict hypotensive events in the following 10–60 minutes (excluding the current blood pressure measurement), we trained a GLMM using RBV as the main predictor. When splitting the data by patient, the model performed well on the training set (AUC = 0.835) but showed poor generalizability to unseen patients (test AUC = 0.62), likely due to substantial inter-individual heterogeneity (random intercept SD = 1.67). To better simulate clinical deployment, where the model could be applied to future sessions from the same patients, we used a session-based split. This significantly improved external performance (test AUC = 0.77). The model achieved a sensitivity of 74%, specificity of 75%, and negative predictive value of 94%. We also evaluated a simplified binary model using the indicator variable “RBV below the dynamic threshold” as the sole predictor. This model yielded similar results (test AUC = 0.775, sensitivity 75%, specificity 73%).

To enhance the generalizability of our predictive model to new patients, we first assessed each variable of interest individually using the previously described modeling approach (Supplementary Table 1). Subsequently, we developed multivariable models that incorporated RBV together with its temporal derivative (dRBV/d*t*), SBP, its decrease from baseline (ΔSBP), and heart rate (HR) (Table 4). The model achieved an AUC of 0.848 on the external test set, with a sensitivity of 74% and a specificity of 81% at the optimal threshold. Using RBV below the dynamic threshold (Table 4) the model also achieved an AUC of 0.848 on the test set, with slightly improved sensitivity (76%) while maintaining good specificity (80%). To benchmark performance against a nonlinear machine learning approach, we trained a regularized XGBoost classifier using cross-validation stratified by patient. On the independent test set, the model achieved an area under the ROC curve (AUC) of 0.862. It reached a sensitivity and specificity of 79%, and a negative predictive value of 96%. The relative contribution of the 20 best predictive variables is illustrated in Supplementary Fig. 1.

**Table 4:** GLMM of the risk of IHD in the next 10–60 min.

Variable	Model 1OR [95% CI]	*P* value	Model 2OR [95% CI]	*P* value
RBV	0.98 [0.96–0.99]	.0178	–	–
RBV below threshold	–	–	1.68 [1.16–2.44]	.0058
dRBV/d*t*	0.26 [0.10–0.70]	.0047	0.26 [0.10–0.70]	.0044
SBP	0.95 [0.94–0.96]	<.001	0.94 [0.93–0.95]	<.001
ΔSBP	1.02 [1.01–1.03]	<.001	1.02 [1.01–1.03]	<.001
HR	1.02 [1.00–1.03]	.0116	1.02 [1.00–1.03]	.0064

Models include a random effect for patient. ΔSBP, difference between initial SBP and current SBP.

## DISCUSSION

In this multicenter study, we evaluated continuous RBV monitoring for assessing IDH risk. RBV was strongly associated with IDH occurrence, and we identified a time-dependent threshold below which the risk of IDH more than doubled. However, RBV alone was insufficient for accurate real-time prediction. The highest predictive performance was obtained when models accounted for patient-specific effects (GLMMs with patient as a random effect) or incorporated additional clinical variables, particularly previous blood pressure measurements.

Intravascular volume loss during hemodialysis reflects the balance between ultrafiltration and plasma refilling, which depends on true volume status and dry-weight accuracy. We compared ultrafiltration volume and dry-weight discrepancy across clusters. As expected, high-risk clusters, with lower RB, had higher ultrafiltration volumes and dry-weight underestimation. These sharper RBV drops likely arise from a combination of dialysis prescription factors and patient-specific determinants of plasma refilling capacity [29, 30].

Andrulli *et al*. [24] reported that RBV at the time of symptomatic hypotension did not differ from RBV at the same dialysis time points in hypotension-free sessions. By contrast, in our study, RBV was lower during IDH compared with periods of hemodynamic stability (Fig. 1b). Andrulli *et al*. also showed that RBV curve irregularity and early steep declines predicted IDH better than RBV level alone, but their findings are limited by a nonstandard IDH definition and lack of external validation. Barth *et al*. [12] evaluated individualized RBV thresholds but found wide variability in the RBV level associated with hypotension. Their mean critical RBV (88%) matched the final value of our RBV threshold. This value also approximates the lower limit of the RBV interval (86%) associated improved survival [31]. Of note, both low and high RBV values were associated with increased mortality, potentially reflecting an increased risk of IDH and fluid overload, respectively. So, patients should not be left fluid overloaded to prevent IDH.

We developed a GLMM-based predictive model that incorporates time-varying RBV and accounts for inter-patient variability through random effects. Trained on previous sessions, it accurately predicted hypotensive events in subsequent ones. In the near future, integrating such a model into dialysis software using prior session data could provide real-time, patient-specific risk estimates and support timely preventive interventions. Practically, an RBV falling below the risk threshold should prompt reassessment of dry weight and reduction of the ultrafiltration rate needed to reach it: by limiting interdialytic weight gain, avoiding net sodium gain, and extending or increasing dialysis sessions. By contrast, simply lowering UF rates during fixed sessions may promote chronic fluid overload.

The relevance of RBV for assessing a patient’s blood volume status has been debated [32]. Kron *et al*. [33] proposed a method to estimate the initial absolute blood volume (ABV). Despite some encouraging preliminary results, a recent study reported poor predictive performance of ABV (AUC = 0.624), when used alone, for predicting IDH [34]. Therefore, irrespective of the technique used, blood volume status alone appears insufficient to predict hypotension risk, consistent with prior evidence that hemodynamic and adaptive responses to hypovolemia vary markedly between patients.

More recently, machine learning tools have been proposed to predict the occurrence of IDH [35, 36]. They have the advantage of inherently modeling complex, nonlinear relationships and potential interactions between variables. Model performance has been primarily evaluated using accuracy. However, high accuracy may reflect a model that favors the majority (non-hypotensive) class. It is essential to define a classification threshold that achieves the best trade-off between sensitivity and specificity to ensure reliable detection of IDH. Some recent high-performing real-time IDH models use the latest blood pressure value to predict hypotension within the next 60 minutes. [37, 38]. This approach may identify events that are already ongoing. Our own XGBoost model reproduced these performances. However, in such cases, it is likely too late for meaningful intervention. Moreover, GLMMs achieved similar performance to XGBoost (AUC = 0.848 vs. 0.862), echoing the findings by Lee *et al*. [37], where logistic regression showed comparable AUC to deep learning.

Our study has several limitations. First, the dataset is of modest size, comprising 56 patients and 459 dialysis sessions. To ensure a sufficient number of events for robust model development and evaluation, we specifically selected patients with a history of frequent IDH episodes. Such a predictive tool would be most relevant for patients who have recently experienced IDH. Second, RBV estimation is based on hematocrit measurements, which can be affected by technical factors such as vascular access recirculation. This could lead to artificially low RBV values in some sessions. However, such artifacts are inherent to all RBV-based systems and reflect the real-life variability of measurements in clinical settings. Third, although the chosen prediction horizon of 10–60 minutes was intended to provide sufficient time for clinically actionable interventions, real-time integration within routine dialysis workflows has not yet been evaluated. Nevertheless, our approach is technically compatible with existing clinical infrastructure, which supports the feasibility of subsequent interventional studies.

RBV monitoring provides valuable insight into patient-specific risk for IDH, provided that inter-individual variability is adequately accounted (e.g. using GLMMs). Future studies should explore the real-time integration of such predictive models into routine dialysis workflows. Coupling these models with active biofeedback interventions, such as adjustments in dialysate sodium concentration or ultrafiltration rate, while ensuring preservation of overall fluid volume status, could facilitate personalized preventive strategies against IDH.

## Supplementary Material

sfag052_Supplemental_File

## Data Availability

Data underlying this article will be shared on request to the corresponding author, in accordance with institutional and ethical guidelines.
