# Supplementary material for: Continuous monitoring of relative blood volume allows real-time assessment of intradialytic hypotension risk
Source: Clin Kidney J. 2026 Feb 19;19(4):sfag052. doi: 10.1093/ckj/sfag052 (PMC13100653; doi:10.1093/ckj/sfag052)
Supplement: sfag052_Supplemental_File [file sfag052_supplemental_file.docx]

**Supplementary Data**

**Detailed Statistical Analysis**

All statistical analyses were conducted using R software version 4.5.0

- Descriptive statistics and univariate comparisons

Continuous variables were summarized as mean ± standard deviation (SD) or median [interquartile range (IQR)], depending on distribution assessed by the Shapiro–Wilk test. Categorical variables were reported as counts and percentages. Relative blood volume (RBV) values were categorized into 5% intervals. For each interval, we calculated the proportion of measurements associated with intradialytic hypotension (IDH) along with exact binomial confidence intervals. The frequency of IDH across RBV categories was compared using Fisher’s exact test. Differences in RBV values between hypotensive and non-hypotensive periods were assessed using the Wilcoxon rank-sum test.

- Association between RBV and IDH

Associations between RBV and IDH were analyzed using generalized linear mixed models (GLMMs) with a binomial distribution and logit link, fitted by maximum likelihood. All RBV measurements were time-aligned with concurrent blood pressure values and IDH status, allowing assessment of a cross-sectional association between RBV and IDH. A patient-level random intercept was included to account for repeated measures. Adding session as an additional random effect did not improve model fit and was therefore not retained in the final model.

- RBV trajectories clustering

RBV trajectories were first interpolated onto a uniform 5-minute time grid spanning 0 to 240 minutes to ensure comparable time series across all hemodialysis sessions, despite differences in measurement frequency or session duration. Unsupervised clustering was then performed at the session level using a partitional time-series clustering framework based on dynamic time warping (DTW), which aligns trajectories with similar shapes even when temporal shifts occur. The DTW distance was combined with a centroid update step using Dynamic Time Warping Barycenter Averaging, as implemented in the dtwclust R package, to obtain representative mean trajectories for each cluster. A three-cluster solution was retained because it represented the minimal number of clusters that produced groups with significantly different risks of IDH. Sessions were assigned to one of three RBV clusters, which were subsequently aggregated into high- and low-risk groups according to observed IDH frequency. Associations between RBV cluster membership and IDH occurrence at any time during the dialysis session were further assessed using logistic GLMMs including a patient-level random intercept to account for repeated measures. The temporal distribution of IDH events was examined by comparing event frequencies at each 30-minute interval across clusters using Fisher’s exact test.

- Time-dependent RBV threshold derivation

At each timepoint, the RBV threshold that maximized the Youden index for discrimination between high- and low-risk clusters was identified. This threshold curve was smoothed using locally estimated scatterplot smoothing (LOESS) to ensure temporal continuity. The 95% confidence intervals of the dynamic RBV threshold were estimated using a bootstrap procedure (500 resamples). For each resample, a LOESS model was fitted and predicted across all time points, and the 2.5th and 97.5th percentiles of the resulting distribution were used as the confidence bounds. The relationship between crossing below the dynamic RBV threshold and concurrent IDH occurrence was assessed using logistic GLMMs with a patient-level random intercept.

- Prediction models

Models were used to predict IDH within 10–60 minutes after a given timepoint (excluding the current BP measurement). GLMMs were fitted using either continuous RBV or a binary indicator for threshold crossing as fixed effect and patient as random effect. Additional predictors of interest were evaluated individually in the same modeling framework. A multivariable logistic GLMM was then constructed including RBV, its temporal derivative (dRBV/dt), systolic BP (SBP), diastolic BP (DBP), and heart rate (HR) as fixed effects and a patient-level random intercept.

Two validation schemes were used:

1. Patient-level split: training on a subset of patients and testing on unseen patients. Patients were stratified into five risk strata based on the proportion of sessions containing an IDH event (event_future), using quintile-based stratification. Within each stratum, a 75% random sample of patients was allocated to the training set, and the remaining 25% to the test set. All sessions from a given patient were kept within the same set.
2. Session-level split: training on early sessions and testing on later sessions from the same patients. Sessions were first classified as positive or negative depending on whether they contained at least one IDH event. Within each class, 75% of sessions were randomly assigned to the training set and 25% to the test set, ensuring that at least one positive session was preserved in each set when possible.

For patient-level validation, GLMM predictions on the test set were generated using fixed effects only, with the patient-specific random effect set to zero, as it cannot be estimated for new patients

Performance metrics included the area under the ROC curve (AUC), sensitivity, specificity, and predictive values. Optimal classification thresholds were determined using the Youden index.

For benchmarking, a regularized gradient boosting model (XGBoost) was used. We selected XGBoost for the predictive modelling because it performs particularly well on structured clinical data, captures non-linear relationships, incorporates regularization to prevent overfitting, and handles missing data natively. These properties make it especially suitable for dialysis monitoring data, where interactions between hemodynamic variables are complex. For model training, we incorporated all clinically relevant predictors, including both temporal (dynamic) and static features. Hemodynamic parameters included systolic blood pressure , diastolic blood pressure, heart rate, the absolute systolic blood pressure drop, and baseline systolic pressure. Relative blood volume–related features consisted of the RBV value and its first and second derivatives. Dialysis-related variables included dialysate sodium concentration, online conductivity–derived sodium, substitution volume rate, session time, pre-dialysis weight, and BCM-derived dry weight. Laboratory and patient characteristics incorporated into the model were hemoglobin, serum albumin , age, dialysis vintage, diabetes status, and the number of antihypertensive medications.

Missing numeric values were imputed by the mean, categorical variables were one-hot encoded, and all variables were normalized. Model training used five-fold cross-validation stratified by patient. Hyperparameters were:

- learning rate: 0.01
- maximum tree depth: 7
- subsample: 0.8
- colsample_bytree: 0.8
- min_child_weight: 10
- gamma: 3
- lambda: 2
- alpha: 0.3

Early stopping was applied to select the optimal number of boosting iterations based on cross-validated AUC. Final performance was evaluated on an independent test set, with ROC curves, AUC, and classification metrics computed using the optimal Youden threshold. Feature importance scores were extracted to quantify the relative contributions of dynamic versus static predictors.

**Supplementary Table 1.** **Odds ratios (OR) and 95% confidence intervals (CI) of variables of interest for predicting intradialytic hypotension within the next 60 minutes, using a generalized linear mixed model (GLMM) with patient as a random intercept.**

| Variable | OR [95% CI] | p-value |
| --- | --- | --- |
| SBP | 0.9332 [0.9239–0.9426] | 7.45e-42 |
| ΔSBP | 1.0441 [1.0370–1.0513] | 4.50e-35 |
| DBP | 0.9367 [0.9228–0.9509] | 1.38e-17 |
| RBV | 0.9568 [0.9447–0.9691] | 1.17e-11 |
| Time | 1.0042 [1.0026–1.0059] | 5.76e-07 |
| Kt/V (ionic dialysance) | 1.8161 [1.3935–2.3669] | 1.01e-05 |
| Heart Rate | 1.0266 [1.0138–1.0396] | 4.14e-05 |
| dRBV/dt | 3.4108 [1.5872–7.3294] | 1.67e-03 |
| Number of antihypertensive treatments | 0.4184 [0.2221–0.7883] | 7.02e-03 |
| Overhydratation | 0.8394 [0.7332–0.9610] | 1.12e-02 |
| ΔNa | 1.0890 [0.9972–1.1893] | 5.77e-02 |
| Dialysate bicarbonate | 1.2675 [0.9823–1.6354] | 6.83e-02 |
| d^2^RBV/dt^2^ | 13003.2600 [0.2336–723789200.0000] | 8.93e-02 |
| Dialysate Na | 0.7590 [0.5407–1.0655] | 1.11e-01 |
| LVEF | 0.9593 [0.9071–1.0144] | 1.45e-01 |
| Hemoglobin | 0.7169 [0.4559–1.1272] | 1.50e-01 |
| Serum Na | 1.1090 [0.9531–1.2905] | 1.81e-01 |
| Dialysis vintage | 1.0008 [0.9996–1.0019] | 2.13e-01 |
| Serum Na (ionic dialysance) | 1.0512 [0.9683–1.1412] | 2.33e-01 |
| Predialysis Weight | 0.9852 [0.9569–1.0144] | 3.18e-01 |
| Postdilution flow rate | 0.9990 [0.9970–1.0011] | 3.46e-01 |
| FTI | 0.9750 [0.9039–1.0516] | 5.12e-01 |
| Albumin | 1.0352 [0.9322–1.1495] | 5.18e-01 |
| LTI | 0.9807 [0.8816–1.0909] | 7.19e-01 |
| Ultrafiltration Volume | 1.0408 [0.8320–1.3020] | 7.26e-01 |
| Age | 0.9925 [0.9439–1.0436] | 7.70e-01 |

ΔNa: difference between serum sodium (ionic dialysance) and dialysate sodium; ΔSBP: difference between initial systolic blood pressure and current systolic blood pressure; DPB: diastolic blood pressure; FTI: fat tissue index; LEVF: left ventricular ejection fraction; LTI lean tissue index; RBV relative blood volume; SBP systolic blood pressure. Overhydration is difference between predialysis weight and dry weight determined by bioimpedance spectroscopy.

**Supplementary Figure 1.**


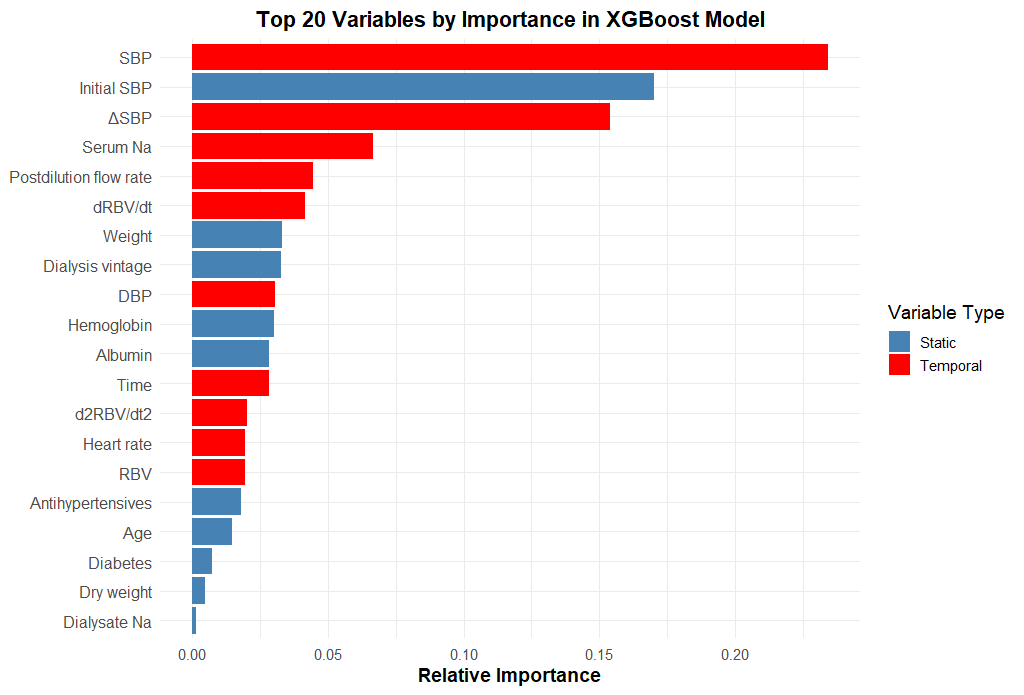


ΔSBP: difference between initial systolic blood pressure and current systolic blood pressure; DPB: diastolic blood pressure; RBV relative blood volume; SBP systolic blood pressure.

**Glossary of Methods and Definitions**

***Bootstrap Confidence Intervals (95% CI)***

A resampling-based method used to estimate the uncertainty around the LOESS-smoothed threshold curve. Multiple bootstrap samples were used to compute the 2.5th and 97.5th percentiles of the predicted thresholds.

***Barycentric Averaging***

A method used as the centroid definition in DTW clustering. DBA computes an average trajectory that best represents all curves within a cluster.

***Dynamic Time Warping based Clustering***

An unsupervised algorithm that groups similar time-series together based on DTW distance a measure of similarity between two time series that aligns them in time so they can be compared even if they evolve at different speeds or rhythms. Sessions with similar RBV patterns over time are assigned to the same cluster. This helps identify distinct hemodynamic phenotypes during dialysis.

***LOESS Smoothing (Locally Estimated Scatterplot Smoothing)***

A nonparametric regression technique used to smooth noisy data by fitting local weighted polynomials. LOESS was applied to the raw RBV thresholds to obtain a continuous, physiologically coherent curve.

***Generalized Linear Mixed Model (GLMM)***

A statistical model extending logistic regression by adding random effects. It accounts for repeated measures within patients. GLMMs were used to estimate the association between RBV (continuous or threshold-based) and the risk of IDH.

***RBV Dynamic Threshold***

A time-varying RBV value computed at each minute to optimally separate the low-risk RBV cluster from the high-risk clusters. The threshold is derived by maximizing the Youden index of time-resolved ROC analyses.

***XGBoost (Extreme Gradient Boosting)***

A machine-learning algorithm particularly suited for tabular clinical data. XGBoost builds an ensemble of decision trees, optimizes model accuracy through gradient boosting, and incorporates regularization to prevent overfitting. It was used to develop multivariable prediction models for intradialytic hypotension.
